# Bladder Care in Palliative Care Inpatients: A Prospective Dual Site Cohort Study

**DOI:** 10.1089/pmr.2020.0060

**Published:** 2020-10-30

**Authors:** Riona Pais, Philip Lee, Shamira Cross, Val Gebski, Rajesh Aggarwal

**Affiliations:** ^1^ Department of Palliative Medicine, Royal Prince Alfred Hospital, Sydney, New South Wales, Australia.; ^2^ Department of Supportive and Palliative Medicine, Crown Princess Mary Cancer Centre, Westmead Hospital, Sydney, New South Wales, Australia.; ^3^ Department of Radiation Oncology, Crown Princess Mary Cancer Centre, Westmead Hospital, Sydney, New South Wales, Australia.; ^4^ Department of Palliative Medicine, Bankstown Hospital, Sydney, New South Wales, Australia.

**Keywords:** bladder care, palliative care, urinary catheterization, urinary symptoms

## Abstract

***Background:*** Urinary catheterization is often undertaken to relieve distressing bladder symptoms in palliative care.

***Objective:*** The primary aim of this study was to determine the incidence of, and clinical indications that predispose patients admitted to palliative care units to, urinary catheterization. The secondary aims were to determine causal factors, including the type of malignancy, antecedent medications, and duration of admission in these patients.

***Methods:*** This was a prospective observational dual site cohort study in palliative care inpatients. Univariate categorical chi-square analysis was performed to compare patients with and without urinary catheterization, and to identify risk factors associated with urinary catheter use.

***Results:*** The incidence of catheterization in this cohort was 41% (43/104) and urinary retention (63%) was the most common cause. Agitation (47%) and urinary incontinence (70%) were common symptoms in those catheterized. Medications that were significantly associated with the need for urinary catheterization were benzodiazepines (*p* < 0.01) and antipsychotics (*p* = 0.01). All measures that define poor functional status were found to be significant (*p* < 0.01). Patients with prolonged hospitalization of greater than three weeks were catheterized more frequently (*p* = 0.017). The majority of patients catheterized (79%) were admitted for terminal care.

***Conclusions:*** The high incidence of urinary catheterization highlights the need for good bladder care for all patients in the palliative care setting. Patients with risk factors include the use of antipsychotics and benzodiazepines, declining functional status and prolonged hospital admission are more likely to be catheterized.

## Introduction

Urinary symptoms are common in palliative care patients and managing urinary symptoms at end of life should be given equal priority as managing distressing symptoms such as pain and dyspnea.^1^ Previous palliative care studies that looked into bladder care and urinary catheterization have estimated that 57%–71% of palliative care inpatients have required urinary catheterization compared with general medical wards where catheterization rates are around 12%–25%.^1–4^ Studies that looked into rates of high and inappropriate urinary catheterization in nonpalliative care patients have cited certain classes of medications, poor functional status, and incontinence as risk factors.^2,5^ However, urinary catheterization does have a beneficial role in ensuring dignity, comfort and convenience in a palliative care setting. There have been no recent studies in palliative care patients who have studied the risk factors that lead to urinary catheterization.

The primary aim of this study was to determine the incidence of, and clinical indications that predispose patients admitted to palliative care units to, urinary catheterization. The secondary aims were to determine causal factors, including the type of malignancy, antecedent medications, and duration of admission in these patients.

## Methods

### Ethics statement

The prospective study was conducted with the approval of the ethics committee of the Western Sydney Local Health district (Approval Number (4056) LNR/14/WMEAD/284).

### Data collection

The study was conducted at two palliative care units located in the Western Sydney Local Health District, New South Wales, Australia. Ethics and governance approval was obtained. All patients admitted to these palliative care units between September 2014 and January 2015 were recruited after written informed consent. Patients with preexisting indwelling catheters were excluded. Recruited patients were interviewed with regard to their urinary symptoms before catheterization for those who were catheterized, and during their admission for those not catheterized, and hospital medical records were reviewed.

#### Catheterization data

Catheterization was performed according to published hospital policy and included the following indications: urinary retention with a postvoid residual volume of 500 mL; patient comfort—either physical discomfort related to frequent changes of clothes and bed linen or the psychological distress associated with incontinence; convenience or ease of care for patients who were either bed bound or had significant difficulty in mobilizing; and distressing symptoms such as incontinence or lower abdominal pain secondary to a distended bladder. Any complication after catheter insertion, such as infection, bleeding, and blockage were recorded as a “catheter-related event.”

#### Functional data

Functional characteristics was assessed using four validated clinical tools—Australian Karnofsky Performance Score (AKPS),^6^ Resource Utilisation Groups–Activities of Daily Living (RUG-ADL),^7^ Palliative Care Phase (Palliative Care Australia),^8^ and the Waterlow score.^9^ Data were recorded on the day of catheterization for those catheterized and on the day of discharge/death for those not catheterized.

#### Medication data

Medication data were collected for four main classes of medication—opioids, anticholinergic medications, benzodiazepines, and antipsychotics. For catheterized patients, the data were obtained in the 24-hour period before catheterization. For patients who were not catheterized, the data were obtained 24 hours before the patient being discharged or death. All opioid doses were converted into oral morphine equivalent daily dose (OMEDD) expressed in milligrams using a clinically accepted dose conversion table.^10,11^ We divided the OMEDD into low (<60 mg), moderate (60–299 mg), high (300–599 mg), and very high doses (>600 mg).^12,13^ We also included medications that had an anticholinergic load according to the Clinician-Rated Anticholinergic Drug Scale.^14^

### Data analysis

As this is a prospective pilot design with the primary objective investigating the determinants of catheterization, formal sample size calculations were not performed. The main focus of the sample size would be to (1) have sufficient number of patients and (2) a reasonable event rate. These considerations suggest that a sample size of 100 or greater would be adequate given an expected catheterization of 50% or higher.

Statistical analyses were performed using the Statistical Package for the Social Sciences and Analysis of Censored and Correlated Data software. The chi-square test was used to compare patients with and without a urinary catheter and to examine association of baseline risk factors with urinary catheter use. Univariate comparisons for other factors (medication use and functional status) are presented as relative risks (RRs) together with 95% confidence intervals (CIs). The level for statistical significance is set at 0.05 and, as this is an exploratory study, there has not been any adjustment for multiple comparisons.

## Results

For a five-month period, 106 consecutive patients were recruited. One hundred and four were included in the study and two participants were excluded due to preexisting urinary catheters. Baseline patient characteristics are provided in Table 1. The majority of the study population (91%) had malignancy as their primary life limiting diagnosis, with the most common being colorectal and lung cancer (16%). Genitourinary cancers were only present in 12% of patients.

**Table 1.** Baseline Patient Characteristics

Total No. of Patients	n (%)
Acute palliative care ward	104
Palliative care unit	55 (53)
Male	49 (47)
Mean age (years)	54 (52)
Admission from	70.5 (23–95)
Home	72 (69)
Hospital	31 (30)
Residential aged care facility	1 (1)
**Primary disease—malignant**	**95 (91)**
CR	18 (16)
Lung	17 (16)
GU	12 (12)
UGI	11 (14)
H&N	10 (10)
Breast	9 (9)
Hematological	6 (6)
CNS	3 (3)
Skin	3 (3)
Others	2 (2)
**Nonmalignant**	**9 (9)**
End-stage kidney failure	2 (2)
End-stage heart disease	2 (2)
End-stage lung disease	2 (2)
Acute surgical condition	2 (2)
End-stage liver failure	1 (1)

Bold values denote the aggregate of primary disease-malignant and nonmalignant.

CNS, central nervous system; CR, colorectal; GU, genitourinary; H&N, head and neck; UGI, upper gastrointestinal.

### Incidence and indications of urinary catheterization

The incidence of catheterization in this study was 41% (*n* = 43) of whom 49% (*n* = 21) were men. All patients were catheterized with indwelling urinary catheters. The majority of patients catheterized (58%, *n* = 25) were from the acute palliative care ward. The median age of those who were catheterized was 69 years.

The most common indication for urinary catheterization was urinary retention (63%, *n* = 27), followed by distressing urinary symptoms such as incontinence or lower abdominal pain (51%, *n* = 22), ease of care (28%, *n* = 12), and patient comfort (19%, *n* = 8). Fifty-one percent (*n* = 22) of patients who were catheterized had more than one recorded indication for urinary catheterization. Distressing urinary symptoms, which included lower abdominal pain, incontinence, and inability to void, was reported by 51% (*n* = 22) of patients. Agitation/confusion (47% vs. 7%), urinary incontinence (70% vs. 7%), and lower abdomen pain (33% vs. 10%) were highly prevalent in those catheterized when compared with those who were not catheterized.

Urinary incontinence (70%, *n* = 30) was the most common symptom among those catheterized. The types of incontinence requiring catheterization were delineated into four main categories—functional, overflow, mixed (urge and stress), and combination (mixed and functional). The majority of patients had either combination incontinence (40%, *n* = 12) or overflow incontinence (37%, *n* = 11). Of note, 33% (*n* = 10) of those reporting urinary incontinence also developed acute urinary retention requiring catheterization.

### Medication risk factors

A comparison of the different classes of medications was made between the catheterized and noncatheterized groups to determine whether there were any medication-related risk factors associated with an increased incidence for urinary catheterization (Table 2). The use of benzodiazepines (*p* < 0.01) (lorazepam, midazolam, temazepam, and clonazepam) and antipsychotics (*p* = 0.01) (haloperidol and levomepromazine) were significantly associated with the need for urinary catheterization.

**Table 2.** Medications Associated with the Need for Urinary Catheterization

Medications	RR [95% CI]	p
Opioids	1.74 [0.635–4.762]	0.28
Benzodiazepines	1.80 [1.211–2.64]	<0.01
Antipsychotics	1.58 [1.1179–2.22]	0.01
High-risk anticholinergic load	1.15 [0.883–1.5154]	0.29

CI, confidence interval; RR, relative risk.

**FIG. 1.**
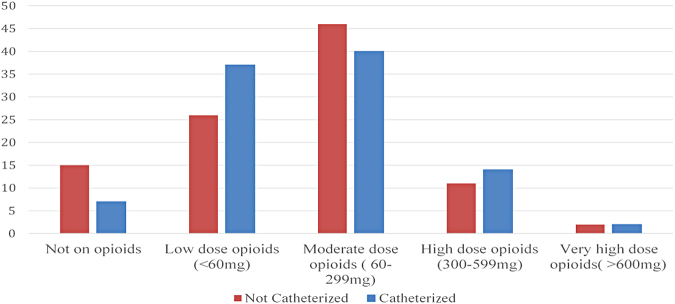
Oral morphine equivalent doses in noncatheterized versus catheterized (%).

**FIG. 2.**
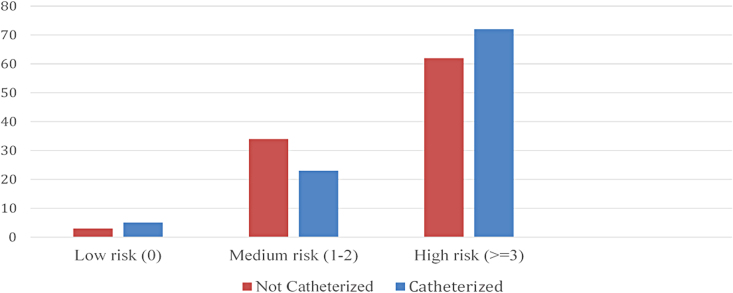
Total anticholinergic risk score in those noncatheterized versus catheterized (%).

There was a nonsignificant trend in the use of opioids and medications with a high anticholinergic load in those having urinary catheterization. Only patients on low amounts of opioids (<60 mg) (Fig. 1) or with a high anticholinergic risk score (>3) (Fig. 2) were more likely to be catheterized. Anticholinergic medications commonly used in this cohort were amitriptyline, glycopyrrolate, hyoscine, and oxybutynin. The median OMEDD in those catheterized was 80 mg compared with 90 mg in those not catheterized. The median OMEDD in those with urinary retention was 90 mg.

### Local causes

**FIG. 3.**
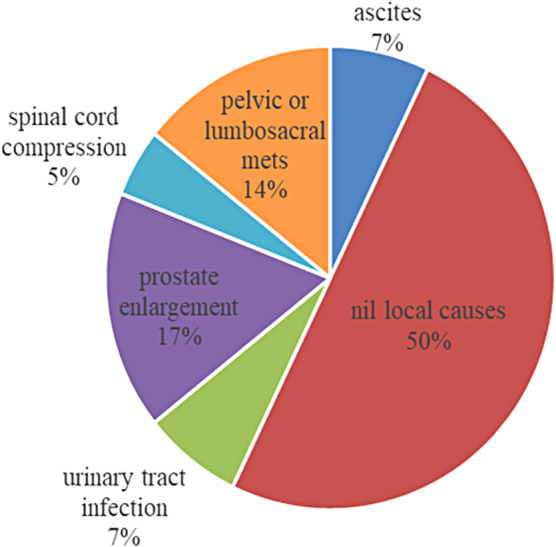
Local factors identified in the catheterized (%).

**FIG. 4.**
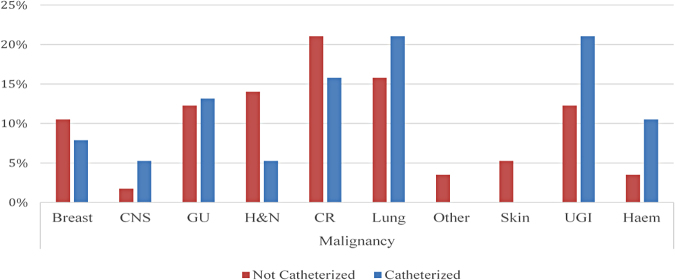
Malignancies in the noncatheterized versus catheterized (%). CNS, central nervous system; CR, colorectal; GU, genitourinary; H&N, head and neck; Haem, hematological; UGI, upper gastrointestinal.

The most common local risk factors identified included benign prostatic enlargement (17%, *n* = 7) and pelvic/lumbosacral metastasis (14%, *n* = 6) (Fig. 3). Although upper gastrointestinal cancers (21%, *n* = 8) and hematological cancers (12%, *n* = 4) were more common in those catheterized, the RR of catheterization associated with both these cancers was 0.987 (95% CI for RR: [0.707–1.377]). Thus, no particular cancer was associated with an increased risk toward catheterization (Fig. 4).

### Functional indices

In patients who were catheterized, 91% (*n* = 39) had a RUG-ADL score of >11, whereas only 43% (*n* = 26) of patients not catheterized had a score of >11. Similarly, 63% (*n* = 27) of those catheterized had an AKPS score of 10–20 compared with only 34% (*n* = 21) in those not catheterized. In those not catheterized, 64% (*n* = 39) had a palliative care phase of 1 or stable compared with only 14% (*n* = 6) catheterized. Twenty-eight percent (*n* = 12) of those catheterized had a Waterlow score of >19 (very high risk) compared with 3% (*n* = 2) of those not catheterized. As shown in Table 3, all these results were statistically significant (*p* < 0.01). The RUG-ADL, AKPS, and Waterlow scores signify that a low functional status was a risk factor for catheterization. The study results also indicate that catheterization was a common intervention to maintain symptom control and quality of life in patients who were unstable, deteriorating, or terminal as indicated by the palliative care phase results.

**Table 3.** Functional Indices Associated with the Need for Urinary Catheterization

Functional Indices	RR [95% CI]	p
RUG-ADL >11	2.12 [1.5662–2.8911]	<0.01
AKPS <30	1.82 [1.2034–2.7643]	<0.01
Palliative care phase 1	0.21 [0.0984–0.459]	<0.01
Waterlow score >19	9.15 [2.1698–38.653]	<0.01

AKPS, Australian Karnofsky Performance Score; RUG-ADL, Resource Utilisation Groups–Activities of Daily Living.

### Mortality rates and length of stay

The overall mortality rate of this cohort was 51% (*n* = 53). The mortality rate in those catheterized was high (79%, *n* = 35) versus (31%, *n* = 19) in those not catheterized. With regard to length of stay, those catheterized remained longer in hospital as 33% (*n* = 14) of those who were catheterized were admitted for greater than three weeks (RR: 2.5, 95% CI [0.185–0.875], *p* = 0.017), whereas in those not catheterized 47% (*n* = 29) were admitted for less than a week. However, 49% (*n* = 21) of catheterized patients were catheterized in their first week of admission.

### Catheterization practices

Bladder scans were done for 65% of patients, before catheterization. All patients with a recorded indication of urinary retention had a bladder scan (*n* = 27). The median bladder scan prompting bladder catheterization was 641 mL with a range of 24–999 mL. The median urine output volume postcatheterization was 500 mL with a range of 20–1400 mL. Adverse catheter-related events, which included bleeding and infection, comprising only 5% (*n* = 2) of total catheterizations. In 11% of patients (*n* = 3), delirium (agitation/confusion) resolved postcatheterization. These patients had a range of 900–1000 mL of urine recorded before catheterization.

Only 4% (*n* = 2) requested that their indwelling urinary catheter be removed and eventually had a successful trial of void. All the remaining catheterized participants remained with an indwelling urinary catheter till their death or discharge with no documented attempts at removing the indwelling urinary catheter. None of the participants who were catheterized received antibiotic prophylaxis.

## Discussion

The incidence of urinary catheterization in this study was found to be 41%. This is comparable with similar studies in the palliative care setting that have shown the incidence vary from 36% to 57% (Refs.^1,3,4^). This is in contrast to studies in the acute hospital setting, where the incidence is much lower, ranging from 14% to 25%.^15–17^

Urinary retention was the most common indication for catheterization, affecting >60% of patients. More than half of the participants in this study had a catheter inserted as a result of distressing symptoms and urinary incontinence was their most common distressing symptom (70%). Studies have shown that urinary incontinence has a negative effect on the social and psychological well-being of patients.^18^ This in turn had a negative impact on their quality of life and dignity and resulted in an increased risk of developing depression.^19^ This study confirms that urinary symptoms are common in the palliative care setting and perhaps identifying these early may help ensure improved bladder care and psychosocial well-being.

Although overflow incontinence requires urinary catheterization, the other types of urinary incontinence, which include urgency, stress, and functional,^20^ can often be managed using multiple noninvasive options such as incontinence pads, diapers, urinary sheaths or condoms, and pouches, as indwelling urinary catheters can also cause pain and discomfort.^21^ This study showed that nearly half of the patients had a urinary catheter inserted for either comfort or convenience.

Owing to increased rates of catheter-associated urinary tract infections and prolonged hospitalization, the current accepted practice is to minimize the use of long-term urinary catheterization in both the general hospital and nursing home populations.^22^ A survey with regard to urinary catheterization in hospitalized adults found that patients prefer noninvasive measures rather than catheterisation.^23^ It is hence recommended that unless the patient has acute urinary retention, measures such as good nursing care, behavioral modification, incontinence diapers, and medication review are the still the basic tenants to achieve good bladder care in most patients. However, indwelling urinary catheters may enhance comfort in those patients who are in the preterminal phase or patients who have perineal or sacral ulcers.^20,24^

Unsurprisingly, certain classes of medications increased the risk of urinary catheterization. Antipsychotics are commonly used to treat both agitation and central nausea.^25,26^ These drugs cause urinary incontinence by causing central dopamine and peripheral alpha blockade, which in turn results in urethral relaxation.^27,28^ Benzodiazepines, which are commonly used for delirium, agitation, or anxiety,^29^ have an effect on the GABA receptors causing relaxation of the striated muscle in the distal urethra and decreased urethral pressure, resulting in urinary incontinence.^30^ A study done in nursing home residents showed that patients on benzodiazepines had a 45% greater risk of urinary incontinence.^31^

This study did not find a statistically significant association between the use of both opioids and anticholinergics, and the increased risk of urinary catheterization. Opioids are thought to cause urinary retention through mu-receptor agonism.^32^ One small study estimated that the incidence of opioid-induced urinary retention was ∼25%.^33^ Medications with a high anticholinergic risk score are thought to reduce bladder contractility resulting in urinary retention.^30^ Previous studies have found a cumulative effect resulting in decreased bladder function in women who were on medications with very high anticholinergic risk scores of >5.^34^ Similar results were found in this study, where the majority of those patients on medications with a high anticholinergic load (anticholinergic risk scores of ≥3) were catheterized.

The common local risk factor for urinary catheterization was benign prostatic hypertrophy and this finding is similar to studies done in hospitalized men presenting with urinary retention where the majority had benign prostatic enlargement.^35^ Increasing functional dependence secondary to debilitation from poor nutritional status and progression of disease was found to be a significant risk factor for urinary catheterization. All the functional indices measured, including a very low AKPS score,^10–20^ high RUG-ADL score (>11), and a high Waterlow score (>19) were associated with an increased risk of catheterization (Table 3). A hospital stay greater than three weeks was also found to be a significant risk factor. Majority of patients catheterized (79%) were admitted for terminal care. These findings reinforce the importance of good bladder care in patients in the preterminal phase of life and the important role that urinary catheterization plays in ensuring dignity, comfort, and convenience.

In this prospective study, very few patients developed complications posturinary catheter insertion (5%). The majority of indwelling catheters were maintained indefinitely with no attempts made at trial of void. This is an area of bladder care where more vigilance on the part of the doctors is needed, not to overlook decisions regarding indwelling catheters. Studies done in nursing homes have indicated that residents with long-term catheters (>28 days) are three times more at risk of receiving antibiotics for catheter-related infections.^36^ Although majority of catheter-related infections are asymptomatic, patients can also develop bladder and kidney stones, and pyelonephritis.^22^ It is important that once acute urinary retention is resolved, a trial of void should be undertaken, especially in those patients not in the preterminal phase.

This was the first prospective study of bladder care in palliative care patients that recruited >100 consecutive participants and looked into patient symptoms and risk/causal factors associated with the need for indwelling urinary catheterization.

### Limitations

Since this was a prospective study, nurses' practices about bladder care may have been affected in those recruited in the trial. Although this was a dual site study, both the sites belong to one health district and the unique population features may have an impact on bladder management practices. A multivariate analysis was not possible due to a small sample size. There were limitations of documentation around using of bladder scans, where a reason for not doing a bladder scan was not listed. There was a lack of documentation with symptoms before catheterization and complications postcatheterization in the preterminal participants and hence there was underreporting of symptoms and complications. There was an overestimation of the effect of urinary catheterization as symptoms postcatheterization was not recorded. The functional scores of participants were affected by individual variability as these were recorded by several doctors and nurses. A large multicentric study to confirm the results from this study, which would also help develop future practical guidelines in the palliative care setting, is required.

## Conclusions

Our prospective dual site observational cohort study involving 104 participants showed that the incidence of urinary catheterization remains high and is a preferred modality to manage distressing urinary symptoms. Routine screening of urinary symptoms is warranted in palliative care inpatients. Significant risk factors for catheterization include the use of antipsychotics and benzodiazepines and a high level of vigilance in patients on these medications is recommended. Urinary catheterization was also strongly linked to declining functional status, poor mobility, cachexia, and prolonged hospitalization. Unless there is an indication of acute urinary retention where a urinary catheter is needed, behavioral measures, nursing care, incontinence diapers, and medication review need to be undertaken to manage urinary problems.

## Abbreviations Used

AKPS: Australian Karnofsky Performance Score
CI: confidence interval
CNS: central nervous system
CR: colorectal
GU: genitourinary
H&N: head and neck
OMEDD: oral morphine equivalent daily dose
RR: relative risk
RUG-ADL: Resource Utilisation Groups–Activities of Daily Living
UGI: upper gastrointestinal
